# Commentary: Greater breast support alters trunk and knee joint biomechanics commonly associated with anterior cruciate ligament injury

**DOI:** 10.3389/fspor.2025.1518946

**Published:** 2025-05-30

**Authors:** Elizabeth Ann Tully

**Affiliations:** Department of Physiotherapy, The University of Melbourne, Parkville, VIC, Australia

**Keywords:** breast, sports bra, knee, biomechanics, ACL, landing, sports

A Commentary on Greater breast support alters trunk and knee joint biomechanics commonly associated with anterior cruciate ligament injury By Fong HB, Nelson AK, Storey JE, Hinton J, Puppa M, McGhee D, Greenwood D and Powell DW (2022). Front Sports Active Living. 4:861553. doi: 10.3389/fspor.2022.861553

## 1 Introduction

This study by Fong et al. investigated the effect on trunk forward lean, knee joint flexion, and knee valgus when female athletes performed a double leg landing task under three conditions. No breast support, low breast support, and high breast support.

The study has flaws with respect to the methodology and the interpretation of the data, so that the authors conclusion ‘Lower levels of breast support are associated with knee joint and trunk biomechanical profiles suggested to increase ACL injury risk’ is questionable.

## 2 Subsections relevant for the subject—limitations of this study

• The *a priori* power analysis called for 14 participants, and only 12 were obtained. A small sample size (acknowledged by the authors)
• The assumption that the participants were wearing the correct sports bra size (acknowledged by the authors)
• High standard deviations for mean knee joint angles, showing that the data are widely spread and there is high variability.
• Conclusions have been made based on statistical significance when actual angle changes measured were very small and likely not clinically significant / meaningful.
• Limitations to the measurement accuracy of the motion capture system, and soft tissue artefact.

## 3 Discussion

### 3.1 Trunk forward lean

Leaning forward when landing protects the ACL, being associated with lower values of the vertical ground reaction force (VGRF), increased flexion of the hip and knee joints, and lower quadriceps activation compared with an upright landing posture (1, 2). Fong et al. have relied on a very small increase (0.7°) in mean trunk forward lean at initial contact, and 0.9° at 100 ms, between the low breast support and high breast support conditions for their proposal that this caused subjects to use a more hip-dominant strategy, thereby putting less stress on the ACL.

### 3.2 Knee flexion

Fong et al. reported very small reductions in mean landing knee flexion angle in the high breast compared to the low breast support condition. A 2.3° flexion reduction at initial contact, and 1.4° at the 100 ms point for the left knee, and less for the right (0.9°). Their interpretation was that this decrease in landing knee flexion with high breast support was more protective of the ACL. It has been demonstrated that decreasing knee flexion on foot contact leads to greater tibial anterior shear force and ACL injury (3–5). The literature suggests that landing knee flexion angles less than 20°-30° put the ACL at risk (6, 7)

At both initial contact and the 100 ms point, standard deviations for mean landing knee flexion angles by Fong et al. were high for both low (±7 L) and high breast support conditions (± 4.7 L). Thus, knee flexion angles were very variable, with subjects in both groups having more flexed and some more extended knees on landing.

Fong et al. also stated that the high breast support condition with decreased knee flexion ‘allowed participants to land with a preferred landing pattern with greater leg stiffness which has been suggested to be indicative of better athletic performance’ (8). In the next paragraph the authors note that increased knee stiffness on landing is accompanied by greater vertical loading rates and VGRF each of which ‘is associated with an increased risk of musculoskeletal injury’ (9) Although landing with reduced knee flexion permits decreased ground contact time and greater execution speed producing a more efficient performance, a stiff landing and strong quadriceps muscle force have been reported as primary contributors to ACL loading (3, 10, 11). It is suggested that there may be an optimal range of knee flexion and stiffness that allows best performance while minimizing risk of ACL injury (8, 11).

### 3.3 Knee Valgus

Fong et al. state: ‘Though the differences in knee valgus angles at 100 ms between breast support conditions were small (−3° −4°), research has suggested that deviations in frontal plane knee joint angle as small as 2° can result in meaningful reductions in the external load required to rupture the ACL’ (5). However, the 2° deviation reported by Chaudhari et al. was measured using a simple frontal plane, three-link passive dynamic model. All joints were constructed as hinge joints, only free to move in vertical and medial-lateral directions.

Chaudhari et al. acknowledge several limitations to their study, including that ‘this model ignores the motion of the leg in the sagittal plane, where most of the motion occurs during normal activity and the muscles exert far greater torques’ (5).

### 3.4 Motion data capture

An advanced 10-camera motion capture system (250 Hz, Qualisys, Goteburg, Sweden) was used to obtain the kinematic data obtained in this study. However, a problem acknowledged by Fong et al. is that kinematic data obtained using a motion analysis system may limit researchers’ findings due to system measurement error and soft tissue artefact (STA) (12, 13) particularly for the very small angular differences reported in this study. For example, a single marker placed over the greater trochanter has been shown to be displaced anteriorly by 17 mm during hip flexion in unresisted pedalling (14) Fong et al. placed rigid clusters of four retroreflective markers bilaterally on the pelvis, thigh, and shank. The use of rigid marker sets on the thigh as opposed to single markers is less prone to STA (15), although an MRI study showed that sagittal knee joint movement (0°–90° flexion) measured from a marker set differed from that of the bones with a maximum of 15° (16). Attached over elastic spandex shorts, Fong et al’s thigh marker sets may have been displaced as the hip was flexed during the landing task.

## 4 Conclusion

It has been shown that good support for the female breasts minimizes discomfort or pain associated with excessive breast bounce during running (17), however it needs a much larger methodologically sound prospective study to determine any link between breast support and ACL injury.
